# Blood-Based Lipidomics Approach to Evaluate Biomarkers Associated With Response to Olanzapine, Risperidone, and Quetiapine Treatment in Schizophrenia Patients

**DOI:** 10.3389/fpsyt.2018.00209

**Published:** 2018-05-25

**Authors:** Adriano Aquino, Guilherme L. Alexandrino, Paul C. Guest, Fabio Augusto, Alexandre F. Gomes, Michael Murgu, Johann Steiner, Daniel Martins-de-Souza

**Affiliations:** ^1^Laboratory of Neuroproteomics, Department of Biochemistry and Tissue Biology, Institute of Biology, University of Campinas, Campinas, Brazil; ^2^Gas Chromatography Laboratory, Chemistry Institute, University of Campinas, Campinas, Brazil; ^3^Mass Spectrometry Applications & Development Laboratory Waters Corporation, São Paulo, Brazil; ^4^Department of Psychiatry and Psychotherapy, University of Magdeburg, Magdeburg, Germany; ^5^Center for Behavioral Brain Sciences, Magdeburg, Germany; ^6^UNICAMP's Neurobiology Center, Campinas, Brazil; ^7^Instituto Nacional de Biomarcadores em Neuropsiquiatria (INBION), Conselho Nacional de Desenvolvimento Científico e Tecnológico São Paulo, Brazil

**Keywords:** schizophrenia, drug response, antipsychotics, lipidomics, biomarkers

## Abstract

This is the first study to identify lipidomic markers in plasma associated with response of acutely ill schizophrenia patients in response to specific antipsychotic treatments. The study population included 54 schizophrenia patients treated with antipsychotics for 6 weeks. Treatment led to significant improvement in positive and negative symptoms for 34 patients with little or no improvement for 20 patients. In addition, 37 patients showed an increase in body mass index after the 6 week treatment period, consistent with effects on metabolism and the association of such effects with symptom improvement. Profiling of plasma samples taken prior to therapy using liquid chromatography tandem mass spectrometry (LC-MS/MS) resulted in identification of 38, 10, and 52 compounds associated with the olanzapine, risperidone, and quetiapine treatment groups, which could be used to distinguish responders from non-responders. Limitations include the retroactive active nature of the study and the small sample size. Further investigations with larger sample sets could lead to the development of a molecular test that could be used to help psychiatrists determine the best treatment options for each patient.

## Introduction

Disease management of acute schizophrenia is achieved by administration of antipyschotics. However, ~40% of patients do not respond adequately to these medications and around 60% end up abandoning treatment due to intolerable side effects (1). As a consequence, the mood-related and cognitive functions of the patients may not improve, making these individuals less capable of functioning adequately in society. The side effects of second-generation (atypical) antipsychotics like olanzapine, risperidone, and quetiapine include metabolic-related responses such as hyperglycaemia, insulin resistance, and weight gain (2). Interestingly, some researchers have suggested that weight gain and other metabolic effects may be linked to the improvement of symptoms (3). These side effects are most likely related to the high affinity of these compounds for other receptor systems in the brain, which are involved in regulation of appetite and food intake (4–6). Such effects can have a deleterious impact on the overall health of patients and can potentially lead to conditions such as insulin resistance, type 2 diabetes, obesity, and cardiovascular complications (7, 8). These issues have led to recommendations for clinicians to closely monitor antipsychotic-treated psychiatric patients for early signs of such side effects (9–11). This has included concerted efforts by researchers from academia and the pharmaceutical industry to identify biomarkers that can be used to predict or monitor treatment responses (8, 12).

These issues have led academic and pharmaceutical industry researchers to embark on biomarker discovery initiatives to help guide treatment decisions and potentially pave the way toward novel therapeutic approaches. A number of molecular studies have already been carried out using multiplex immunoassay profiling of serum/plasma proteins to identify potential relationships between antipsychotic treatment responses and biomarker levels (12–14). However, none of these studies attempted to identify biomarker profiles that are predictive of response in cohorts treated separately with different antipsychotics. This is critical since different patients may show a better response to a distinct drug. In a recent publication, we reported on the analysis of plasma samples from a cohort of 58 schizophrenia patients using shotgun mass spectrometry proteomic profiling (15). Our objective was to identify proteins and protein pathways involved with an effective response to atypical antipsychotics, although, as with the above studies, no attempt was made to identify treatment-specific signatures.

Given the effects on metabolic functions mentioned above, we have carried out a lipidomic profiling study of plasmasamples obtained from the same schizophrenia patients prior to their treatment with the different antipsychotics. The analysis consisted of ultra-performance liquid-chromatography tandem mass spectrometry (UPLC-MS/MS) followed by the multivariate data analysis of the overall lipidomic profiles from all patients simultaneously (16). The main objective was to identify lipid profiles that could be used to predict response to treatment with either olanzapine, risperidone, or quetiapine. This is the first study which has attempted to assign specific biomarker profiles for predicting response to distinct antipsychotics. This study provides the groundwork for development of objective clinical tests that can be used to help psychiatrists in the choice of treatments for individuals presenting with acute schizophrenia in line with personalized medicine objectives.

## Methods

### Samples

The samples had already been obtained from a cohort comprised of 54 acute schizophrenia patients who were treated with either olanzapine (*n* = 17), risperidone (*n* = 23), or quetiapine (*n* = 14) (Supplementary Table 1), as described previously (15). Diagnoses were performed using DSM-IV criteria and the Structured Clinical Interview (SCID-I) (17). Blood samples had been collected by venous puncture in the psychiatric clinic at the University of Magdeburg, Germany, as part of naturalistic study of acutely ill in-patients who were un-medicated for at least 6 weeks prior to inclusion. Citrate plasma samples were prepared and analyzed at a time when the patients were suffering from acute illness [designated time zero (T0)]. Individuals with other medical diseases were not included in the study. After the 6-week treatment period, all samples were grouped according to whether (*n* = 34) or not (*n* = 20) the patients responded favorably to the treatment. A favorable response was defined as a 50% reduction of total Positive and Negative Syndrome Scale (PANSS) scores. These scores were corrected by subtraction of the minimum the scores which represented no symptoms (7, 7, and 16 for PANSS positive, negative and general scores, respectively) (15). Exclusion criteria included substance abuse disorder or symptoms induced by a non-psychiatric medical illness or treatment, including immune diseases, immunomodulatory treatment, cancer, chronic terminal disease, cardiovascular disorders, dyslipidemia, diabetes, and severe trauma. The institutional review board (ethics commission of the University of Magdeburg) approved the study, (process 110/07, from November 26th, 2007 amended on February 11th, 2013). Written informed consent was obtained from all participants.

### Sample preparation

Lipids from 25 μL plasma samples taken at T0 were extracted by addition of 100 μL isopropanol. Samples were vortexed for 1 min and then incubated 10 min at room temperature. The samples were kept at −20°C for 18 h and centrifuged at 14,000g for 20 min. The pellet was discarded and the supernatant dried in a vacuum centrifuge and reconstituted in 100 μL isopropanol (IPA)/acetonitrile (ACN)/water (2:1:1 v:v:v) (18). Ten quality control (QCs) injections were run along with the clinical samples. These were composed by equal aliquots of each of the plasma sample to monitor the preparation and LC-MS/MS performance.

### Liquid chromatography

All chemicals were purchased from Sigma-Aldrich (Seelze, Germany) and were of high performance liquid chromatography (HPLC) grade, or higher, purity. An Acquity H-Class UPLC (Waters Corporation, Milford, MA, USA) was used as the inlet for mass spectrometry. The column used was an ACQUITY UPLC® CSH C18, 2.1 × 100 mm, 1.7 μm particle size (Waters Corporation), operating at a flow rate of 0.4 mL/min and at 55°C. The chromatographic separation was performed in gradient mode using a mobile phase system consisted by two solvents, A and B. Solvent A was ACN/water (60:40, v:v) with 10 mM ammonium formate and 0.1% formic acid, and phase B was IPA/ACN (90:10, v:v) with 10 mM ammonium formate and 0.1% formic acid. The gradient steps started with 60% A and 40% B, changing linearly to 57% A and 43% B in 2.0 min, to 50% A and 50% B in 2.1 min, to 46% A and 54% B in 12.0 min, to 30% A and 70% B in 12.1 min, to reach 1% A and 99% B in 18.0 min and then returning to the initial composition (60% A and 40% B) in 18.1 min. The buffer remained at this composition until 20 min prior to preparing the system for the next injection. The injection volume was 1.0 μL.

### Mass spectrometry

The inlet system was coupled to a hybrid quadrupole orthogonal time-of-flight mass spectrometer, Xevo G-2 XS QT of (Waters Corporation, Manchester, UK), controlled by MassLynx 4.1 software. Data were acquired in positive electrospray ionization (ESI+) with the capillary voltage set to 2.0 kV, cone voltage to 30 eV and source temperature to 150°C. The desolvation gas was nitrogen, with flow of 900 L/h and temperature of 550°C. Data were acquired from m/z 100 to 2,000 in MS^E^ mode, during which the collision energy was alternated between low (2 eV) and high (ramped from 20 to 30 eV). Leucine-enkephalin (Waters Corporation, Milford, MA, USA), C_28_H_37_N_5_O_7_, ([M+H]+ = 556.2771 m/z) was used as lock mass reference at concentration of 0.2 ng/L with flow rate of 10 μL/min.

### Data processing and statistical analyses

Progenesis QI 2.2 (Nonlinear Dynamics, Indianapolis, IN, USA) software was used to process data for peak detection, multivariate analysis and identification. All samples were normalized using the total ionic current. After that, data filtering was performed by removing ions present in blank samples. The ion abundance threshold filtering was also applied, which evaluated the minimal ion abundance that could provide a good precursor ion signal with a consistent isotopic pattern. A final filtering step was made based on retention time by checking sample chromatograms to define the retention time window over which the lipids eluted. After the data filtering steps, the normalization option was changed from total ion current to the summed ion abundance of all compounds. For compound identification, the Lipidmaps database was used, with searching parameters as follows: precursor mass error ≤ 10 ppm, fragment tolerance ≤ 10 ppm. Other parameters such as fragmentation score were also considered for disambiguation.

### Data analysis

The output variables were the ion abundance of the chromatographic signals from the lipids according to their corresponding mass/charge (m/z) obtained from the mass spectrometer. Approximately 1,600 m/z signals with different elution times were detected in total. To identify the appropriate adducts for data processing, a spectral evaluation of different lipid classes and ion abundances were carried out. The selected adducts were [M+H-H2O]+, [M+H]+, [M+Na]+, [M+K]+, [2M+H]+ and [2M+Na]+. The normalized m/z signals were imported into Matlab® R2013b (The Matworks, Natick, MA, USA) as single data matrices Xi(I × J) for each treatment, with i = 1 (risperidone), 2 (olanzapine), or 3 (quetiapine), I = number of samples in the corresponding treatment, and J = number of m/z signals: risperidone = 1,610; olanzapine = 1,632; and quetiapine = 1,628. Multivariate analyses were performed with the autoscaled data using Partial Least Squares-Discriminant Analysis (PLS-DA) and Principal Component Analysis (PCA), employing the Pls Toolbox 8.1 (Eigenvector Research Inc., Wenatchee, WA, USA). One-way ANOVA *F*-Test analyses were performed in Matlab using in-house scripts.

### PLS-DA

The most relevant m/z signals from baseline samples used to distinguish the patients who did or did not respond to the treatments were extracted from each Xi data matrix using a variable selection approach based on double cross-validation (CV2) PLS-DA (19). For CV2, Xi matrices were randomly split into 2 submatrices Xical (A, J) and Xitest (B, J), with A = 17, 12, or 9, and B = 5, 4, or 2, for either risperidone, olanzapine, and quetiapine, respectively. This approach helped to keep the proportion of the overall m/z signals between responders and non-responders in the submatrices similar to the corresponding Xi matrices. PLS-DA was performed only for Xical, using 4-fold Venetian blind cross-validation for model optimization. Xical submatrices were also arranged to allow simultaneous extraction of rows from both responder and non-responder groups of patients during the inner cross-validation process, and the correct number of latent variables (LVs) for the models was obtained in the lowest root mean squares error of cross-validation (RMSECV). After model optimization, only the Variable Importance for Projection (VIP) scores upon a threshold were selected while extracting the most informative m/z signals for the classification from Xical submatrices. This threshold was defined iteratively based on reaching the point below which the PLS-DA models did not increase their RMSECV. This was computed for new models each time a new threshold was established. The overall CV2 procedure was performed 500 times, and only those m/z signals that were selected in at least 80% (risperidone and olanzapine) and 95% (quetiapine) of the iterations were considered relevant for distinguishing between responders and non-responders. The remaining m/z signals were discarded, since the contrary approach resulted in an overall increase of the RMSECV and misclassification of predictions from the Xitest. No additional variable selection step was performed once the Xi matrix contained only the most frequent m/z signals selected previously from the histograms. This approach of combining variable selection into double cross-validated PLS-DA models provided the most important m/z signals from the pooled data without fitting overly-optimistic PLS-DA models. This is due to the fact that the final m/z signals were selected based on non-biased histograms of random iterations and because model performances were strictly evaluated, given that they were computed for independent samples. A similar approach using PLS-DA for analyte selection has been published elsewhere (20). Here we intended to apply a high stringency model, so that only the most robust data are selected. The drawback is that some valid signals may be filtered out.

### One-way ANOVA *F*-test and PCA

The final m/z signals selected from the PLS-DA modeling were ranked individually according to their ability to distinguish between responder and non-responder patients, using one-way ANOVA *F*-test analysis. False discovery rates (FDR) were also estimated for the respective m/z signals according to a null distribution ANOVA *F*-test approach, as described previously in metabolomics studies (21, 22). Next, PCA was performed to highlight the separation between responders and non-responders to each antipsychotic treatment when considering only the selected compounds in which FDR ≤ 0.05 (i.e., probability of type-I error ≤ 5%).

### Data sharing

The ethics committee approval for this project does not include sharing the mass spectrometry raw files obtained here, as these are pertinent information to the participants of the study. Interested researchers should look for us in order to sign a non-disclosure agreement to be submitted to the ethics committee.

## Results

### Patient response to treatment

According to the criteria set in the Methods section, 34 out of 54 total patients showed a good response to treatment (see Supplementary Table 1). There were no significant differences observed for age, illness duration, BMI, gender, smoking, PANSS negative, or PANSS general scores at baseline between the responders and non-responders (Table 1). However, significantly higher PANSS positive scores (*P* = 0.005) were observed at baseline for the responders compared to the non-responders. In addition, 38 out of the 54 patients showed an increase in BMI following the treatment. In the responder group, 27 out of the 34 patients showed an increase in BMI with an average increase of 1.22 ± 1.42 kg/m^2^ (mean ± sd; *n* = 34). In the non-responder group, 11 out of the 20 patients had an increased BMI (0.22 + 1.43; *n* = 20). There difference in the BMI gain across the responders and non-responders was significant (*p* = 0.033; Mann–Whitney test).

**Table 1:** Assessment of baseline variables, comparing patients in the responder (R, *n* = 34) and non-responder (NR, *n* = 20) groups.

		**Median (Q1, Q3)**	***P*-value**
Age (years)	R	36.0 (30.3, 47.3)	0.199^a^
	NR	31.5 (25.0, 43.3)	
Illness duration (years)	R	1.5 (0.0, 9.3)	0.689^a^
	NR	1.5 (0.0, 9.3)	
BMI (kg/m^2^)	R	23.8 (21.6, 29.8)	0.484^a^
	NR	23.5 (22.0, 26.8)	
PANSS positive scores	R	26.0 (16.3, 21.0)	**0.005**^a^
	NR	21.0 (16.8, 24.5)	
PANSS negative scores	R	9.5 (5.0, 18.0)	0.429^a^
	NR	11.5 (6.0, 22.3)	
PANSS general scores	R	27.0 (22.0, 32.8)	0.255^a^
	NR	30.0 (21.0, 39.8)	
Gender (male/female)	R	19/15	0.821^b^
	NR	13/7	
Smoking (yes/no)	R	23/11	0.807^b^
	NR	15/5	

*As the data were unevenly distributed, Shapiro-Wilk tests were use to determine significance values for continuous variables (median and quartiles 1 and 3 shown) and Fisher's exact test was applied for contingency variables. Values which were significantly different are indicated using bold font*.

a H-test;

b *Fisher's exact test*.

### Selection of m/z peaks for best separation of responders and non-responders for each treatment group

**Figure 1:**
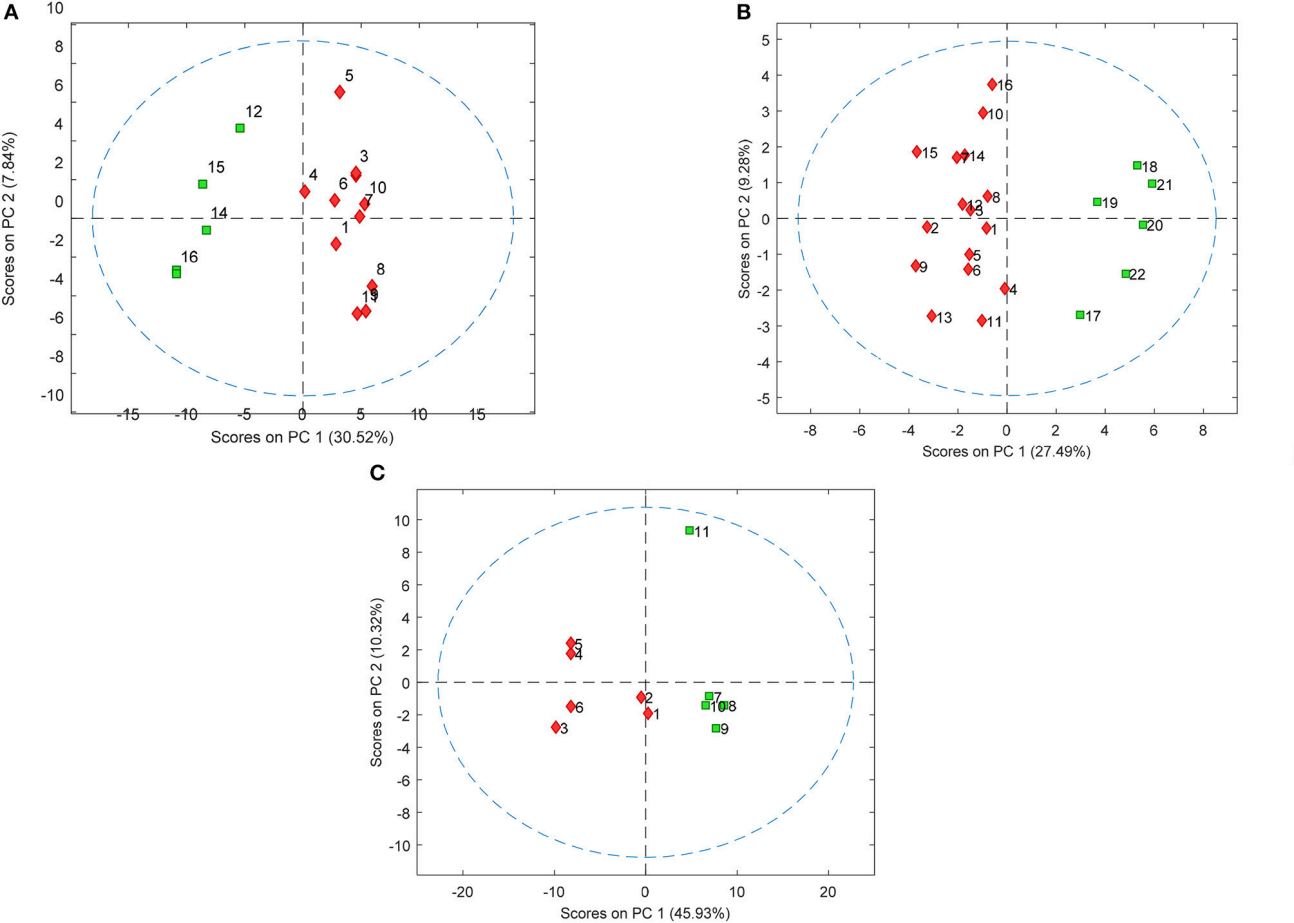
PCA plots showing separation of patients who either responded or did not respond to **(A)** olanzapine, **(B)** risperidone, and **(C)** quetiapine treatment using baseline plasma lipid levels (each symbol represents a patient sample). Responders and non-responders are represented in red and green, respectively.

PLS-DA models were used to extract the most relevant m/z signals from the pooled data for the best separation of responders and non-responders in each treatment group. This resulted in selection of 134, 36, and 119 m/z signals for the olanzapine, risperidone, and quetiapine treatment groups, respectively out of more than 1,600 compounds in total (Table 2). Due to the fact that the PLS-DA extracts linear correlations in the multivariate data to maximize discrimination of the samples in a supervised manner, some false positive signals might be included. For this reason, the ability of each m/z signal to distinguish between responders and non-responders was tested using one-way ANOVA *F*-test analysis combined with FDR estimations. The F-ratio thresholds for estimating the FDR (confidence level ≥ 99%), obtained from the null-distribution ANOVA F-ratio approach, considered all possible null class comparisons in both groups (i.e., response or non-response) of patients, for each antipsychotic treatment group. The compounds for which the FDR was <5% are more likely to be associated to the medication effectiveness. Therefore, these compounds were tentatively identified for the selected olanzapine, risperidone, and quetiapine signals (Supplementary Tables 2–4; non-identified m/z signals are provided in Supplementary Table 5). The PCA plots considering only those m/z signals for which the FDR ≤ 5% resulted in clear discrimination between responders and non-responders for the olanzapine (Figure 1A), risperidone (Figure 1B), and quetiapine (Figure 1C) treatments. These separations were associated with a total (i.e., identified and non-identified) of 66 compounds for olanzapine (Figure 1A), 24 compounds for risperidone (Figure 1B) and 52 compounds for quetiapine (Figure 1C).

**Table 2:** Performance of double cross-validated PLS-DA models after *N* = 500 random iteration (average values and standard deviations in the brackets) to select the main m/z signals while classifying between responders and non-responders for each treatment group.

	**Risperidone**	**Olanzapine**	**Quetiapine**
# of latent variables	1.06 (0.40)	1.23 (0.46)	1.12 (0.33)
RMSECV	0.005 (0.024)	0.04 (0.06)	0.03 (0.05)
Misclassified	0.4 (4.5) %	0.3 (2.3) %	0
M/Z selected	36	134	119

## Discussion

This is the first study to use a lipidomic profiling approach in an attempt to detect lipid-based molecules in plasma samples taken from patients prior to treatment that could be used for prediction of response to specific antipsychotic treatments. The PLS-DA models resulting in low root mean squares error of cross-validation and misclassifications infers robustness (non-biased) of the final selected lipid signals, given that the models correctly classified most of the patients independently throughout the random cross validation iterations. These results, along with the ranking of the signals according to their respective ability to distinguish between responder and non-responder patients, was provided by the one-way ANOVA *F*-test analysis combined with the corresponding false-positive probability estimations. This guided the biological investigation toward only those potential hits closely related to the responses of patients to treatment with the specific antipsychotics. The m/z signals that were not designated as potential hits do not necessarily infer non-relevance to the study. Instead, they have a higher probability (>5%) of being false-positives in these datasets, according to the adopted statistical metric.

The brain is rich in lipids, which act within specific brain regions to regulate processes that can impact complex processes such as behavior, emotions, cognition, and learning (23). Evidence has accrued that phospholipids play an important role in the structure and function of membranes and the associated pathways appear to be impaired in schizophrenia (24). Therefore, counter changes in phospholipids may be associated with the therapeutic response to antipsychotics in schizophrenia patients. Recent developments in the field of lipidomics have allowed characterization of hundreds of individual lipid species in human blood which may be altered in disease states (25). For example, deregulation of cholesterol has been associated with altered levels of some steroid hormones and development psychiatric disorders including autism (26), altered metabolism of sphingolipids such as ceramide has been observed in some cases of depression (27) and patients with psychosis often show subclinical dyslipidemia (28).

Most antipsychotics in current use act as dopamine and serotonin blockers and. the circulating levels of drugs such as risperidone and quetiapine are metabolized in the liver to produce the active metabolites (29) and some classes of lipids can inhibit the activity of the metabolizing enzymes. Altered phospholipid content in the membrane can affect metabolism through the cytochrome P450 family of metabolic enzymes by altering protein conformation and interactions essential for activity (30). Cirulating nutritional factors including lipids can also affect the efficiency of these metabolizing enzymes (31). This may be important as response of schizophrenia patients to antipsychotics may be related to metabolism of these drugs in the liver. For example, olanzapine is normally metabolized to its 10-and 4′-N-glucuronides, 4′-N-desmethylolanzapine [cytochrome P450 (CYP)1A2] and olanzapine N-oxide (flavin mono-oxygenase 3) (32), risperidone is metabolized primarily by CYP2D6 and to a lesser extent by CYP3A4, and quetiapine is known to undergo an N-dealkylation catalyzed by CYP3A4/5 (33). Furthermore, olanzapine is metabolized by direct glucuronidationby CYP1A2 and by CYP2D6 and CYP3A4, although the later two enzymes play a smaller role in this conversion. In addition, quetiapineis metabolized by CYP3A4 although aldehyde oxidase is the enzyme responsible for most of its metabolism (34). Thus, an altered composition of lipid in blood could lead to changes in the levels of some of these metabolites and thereby affect treatment response. Further studies should investigate the effect of specific lipids on metabolism of each of these antipsychotics, given the potential link to treatment response. This study has provided a list of lipids associated with treatment response to olanzapine, risperidone, and quetiapine which could be used as potential starting point in such an investigation.

## Limitations

There are a number of limitations of this study. Firstly, this was a retroactive analysis of samples provided from a previous naturalistic study and there was only a limited sample size for determining effects of each separate drug treatments. Thus, replication is required using a different sample set with larger numbers for each treatment group. Also, patients who showed a good response to the treatment also had the most severe symptoms at baseline, as assessed by the PANSS positive scoring system. Although the small sample size was a limitation of this study from a statistical point of view, the data treatment strategy comprising variable selection and statistical double cross-validation presented here assured the non-biased selection of only the most robust m/z signals that distinguish responders and non-responders groups of patients in the dataset. Finally, the natural heterogeneity of schizophrenia makes identification of biomarkers challenging (35). Further studies should be carried out using patient populations that have been stratified strictly using either clinical or molecular biomarkers. Finally, although the results give high confidence on identification of the lipid class, size of carbon chains and number of double bonds, it was not possible to distinguish between isomer candidates. Specifically, the observed fragmentation helped to define the size and number of double bonds of each carbon chain for many compounds, considering the sn-1 and sn-1-H_2_O (and sn-2 and sn-2-H_2_O for lipids with more than one carbon chain). However, the position of the double bonds could not be determined using the current mass spectrometry approach as this would require comparison with analytical standards. Finally, it is important to highlight that our aim here is to look for a molecular signature to predict antipsychotic responsiveness. As it can be seen in the Supplementary Tables, among the compounds we analyzed, a number of them are unidentified. We are not focusing here in their identification. If these are consistently present in the samples analyzed and are able to separate responders from non-responders, we reached our objective. We are currently working on their identification, in order to describe which of these compounds are triggering molecular pathways associated to an effective antipsychotic response and which are involved with poor response. This data will provide, in the future, biological leads that may be useful for the development of new treatments.

## Conclusions

Previous studies have demonstrated that first onset schizophrenia patients may also show signs of metabolic syndrome (7, 36). This includes elevated levels of insulin-related peptides (37, 38), increased insulin resistance (39, 40) and altered lipid profiles (28) in the blood. This is the first study to identify specific lipidomic signatures for prediction of response to specific antipsychotics. Given further validation of these findings, the signatures could be developed into a rapid assay using a platform such as selective reaction monitoring (SRM) mass spectrometry to aid the process of antipsychotic selection in the treatment of patients with acute psychosis. This fits in with ongoing strategies of translational and personalized medicine for improved treatment outcomes of patients suffering from this debilitating psychiatric disease.

## Author contributions

DM-d-S conceived, organized, and supervised all steps of the study. DM-d-S and PG wrote the paper together with PG. JS collected and provided blood plasma samples and contributed with paper writing. AA prepared plasma samples and ran the mass spectrometry experiments helped by AG and MM. GA performed all statistical analyses supervised by FA. PG helped in data analyses. All authors had access to the final version of the manuscript.

## Conflict of interest statement

AG and MM are employees of Waters Corporation (São Paulo, SP, Brazil). The other authors declare that the research was conducted in the absence of any commercial or financial relationships that could be construed as a potential conflict of interest. Results here led to a patent request in Brazil (BR 10 2017 025852 1 - pending). Documents 110/07 and 67/10.

## Supplementary material

Supplementary Table 1 Patients demographics.

Supplementary Tables 2–4 **List of compounds (FDR was <5%) associated to the medication effectiveness. Non-identified m/z signals are provided in Supplementary Table 5**.

Supplementary Table 5 List of non-identified compounds for each of the medication evaluated.
